# Central mechanisms of muscle tone regulation: implications for pain and performance

**DOI:** 10.3389/fnins.2024.1511783

**Published:** 2024-12-09

**Authors:** Timothy W. Cacciatore, David I. Anderson, Rajal G. Cohen

**Affiliations:** ^1^Independent Researcher, Cheltenham, United Kingdom; ^2^Department of Kinesiology, Marian Wright Edelman Institute, San Francisco State University, San Francisco, CA, United States; ^3^Department of Psychology and Communication, University of Idaho, Moscow, ID, United States

**Keywords:** muscle tone, postural tone, neurophysiology, stiffness, musculoskeletal pain, movement coordination, complimentary and integrative health

## Abstract

Muscle tone represents a foundational property of the motor system with the potential to impact musculoskeletal pain and motor performance. Muscle tone is involuntary, dynamically adaptive, interconnected across the body, sensitive to postural demands, and distinct from voluntary control. Research has historically focused on pathological tone, peripheral regulation, and contributions from passive tissues, without consideration of the neural regulation of active tone and its consequences, particularly for neurologically healthy individuals. Indeed, simplistic models based on the stretch reflex, which neglect the central regulation of tone, are still perpetuated today. Recent advances regarding tone are dispersed across different literatures, including animal physiology, pain science, motor control, neurology, and child development. This paper brings together diverse areas of research to construct a conceptual model of the neuroscience underlying active muscle tone. It highlights how multiple tonic drive networks tune the excitability of complex spinal feedback circuits in concert with various sources of sensory feedback and in relation to postural demands, gravity, and arousal levels. The paper also reveals how tonic muscle activity and excitability are disrupted in people with musculoskeletal pain and how tone disorders can lead to marked pain and motor impairment. The paper presents evidence that integrative somatic methods address the central regulation of tone and discusses potential mechanisms and implications for tone rehabilitation to improve pain and performance.

## 1 Introduction

Chronic musculoskeletal pain, particularly neck and back pain, is the leading cause of time lost to disability worldwide, and it continues to increase (Vos et al., 2017; Stevans et al., 2021). In most cases, an underlying pathoanatomical cause cannot be found (Henschke et al., 2009; Hartvigsen et al., 2018). Various factors have been identified as important to developing and perpetuating chronic pain, including psychosocial factors (Lee et al., 2015) such as fear and low self-efficacy, and the sensitisation of central pain pathways (Nijs et al., 2021). However, the efficacy of treatment for chronic musculoskeletal pain remains inadequate (Webster and Markman, 2014; O'Keeffe et al., 2016; Traeger et al., 2019; Williams et al., 2020). It has been suggested that other mechanisms must contribute to chronic pain (Lee et al., 2015). Indeed, growing evidence suggests that sensorimotor deficits (Tanaka et al., 2021; Alshehri et al., 2024) and related plasticity (Jenkins et al., 2022) can predict the transition to chronicity and may play a causal role in persistent pain.

### 1.1 Muscle tone, pain, and performance

Muscle tone is a ubiquitous and foundational sensorimotor phenomenon, underlying muscular tension and postural support, which has been poorly understood (Ivanenko and Gurfinkel, 2018) and could contribute to pain. Muscle tone has passive and active components. The passive component originates from viscoelasticity of multiple tissues and has been discussed in detail elsewhere (Mense and Masi, 2010; Ivanenko and Gurfinkel, 2018). The active component arises from low-level, sustained muscle activation, which is regulated by complex central and peripheral neural circuits. Pathologies of tone such as hypertonia and hypotonia commonly result in pain (Alwardat et al., 2019; Ostojic et al., 2019; Miller, 2020), and active muscle tone is generally disrupted with musculoskeletal pain (Dankaerts et al., 2006; Mork and Westgaard, 2006; Park et al., 2013; Claus et al., 2018; Worman et al., 2023). Such disruptions in tone would affect joint loading. Active muscle tone is also closely related to excitability, which has been proposed to play a critical role in chronic pain (Hodges and Tucker, 2011; Jenkins et al., 2023). As with pain, pathological disruptions in tone can severely affect motor ability (Goo et al., 2018; Ha and Sung, 2023). Thus, the regulation of healthy tone may be an important target for chronic pain as well as movement coordination (Cacciatore et al., 2014; Chen et al., 2023; Worman et al., 2023; Wendt and Waszak, 2024).

### 1.2 Inadequacy of tone intervention

The past several decades have led to increased understanding of muscle tone, in particular how it is centrally controlled. Active muscle tone is regulated by a complex, dynamically adaptive neural system that is interconnected across the body and highly sensitive to postural demands (Gurfinkel et al., 2006; Takakusaki et al., 2016; Ivanenko and Gurfinkel, 2018). It is closely connected with excitability of lower levels of the nervous system, which is tuned by tonic descending neural drive (Deliagina et al., 2014).

While many practices address tension as a means for improving pain and performance, they generally do so without reference to the complex adaptive brain systems that regulate muscle tone. Instead, interventions often involve isolated stretching and strengthening (Slade and Keating, 2006; Geusebroek et al., 2023), which emphasizes peripheral contributions, or focus on volitionally controlling posture (Harvard Medical School, 2017) or relaxing muscles (Conrad and Roth, 2007). None of these approaches address the complexities of the tone system, which is multifaced and involuntary, and it is not clear how to best address it. However, somatic practices have been found to alter muscle tone (Stephens et al., 2006; Masi and Hannon, 2008; Cacciatore et al., 2011a) and improve pain (Little et al., 2008; MacPherson et al., 2015; Kong et al., 2016; Berland et al., 2022) and motor control (Jones et al., 1959; Cacciatore et al., 2014; Yang et al., 2014; O'Neill et al., 2015; Berland et al., 2022). These methods may give insight into how to influence and improve tone regulation.

The aim of this paper is to convey a modern understanding of the regulation of active muscle tone, address how it may be related to pain and performance, and highlight practices that may yield insight into how to address it.

## 2 What is muscle tone?

Muscle tone relates to the ongoing tension within skeletal muscle that results from passive viscoelasticity and active tonic muscle contraction. Sources of viscoelasticity include connective tissue as well as actin-myosin cross-bridge formation when muscles are held at a static length. This is most commonly seen as short-range stiffness when muscles are active (Rack and Westbury, 1974), but it also occurs in passive muscles held at a fixed length (Proske and Morgan, 1999). We consider both of these factors to be contributors to passive tone as they are not under immediate control of the nervous system, even though short-range stiffness is a property of active muscle. It is important to note that the active component of tone regulation differs from phasic postural control (Ivanenko and Gurfinkel, 2018), such as the transient muscular activation that maintains balance. Bernstein considered tone to be the foundational level of the motor control hierarchy (Latash et al., 1996; Profeta and Turvey, 2018).

### 2.1 Resting vs. postural muscle tone

**Figure 1:**
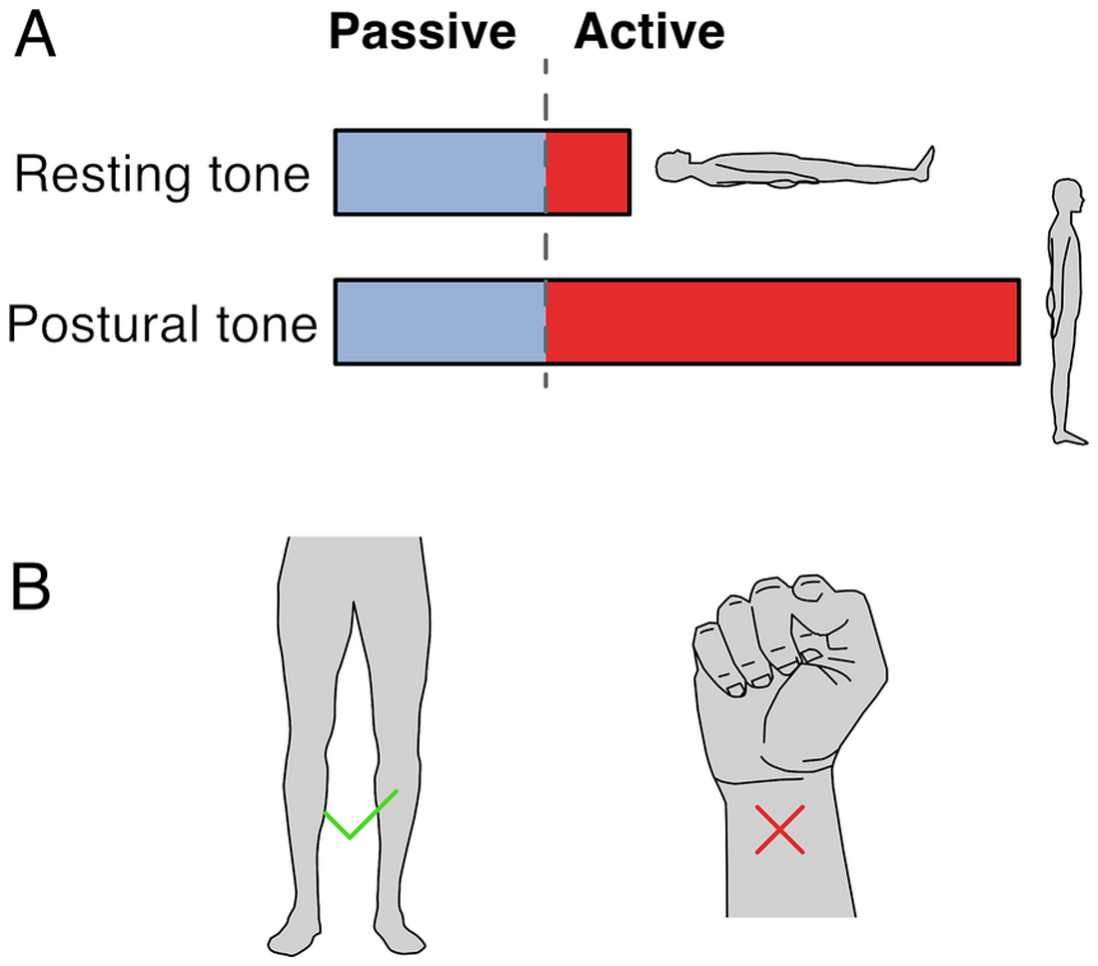
Resting and postural tone. **(A)** Resting tone is assessed with the subject fully supported and is largely passive except for axial and proximal muscles. Postural tone is assessed during active postural maintainence. Resting and postural tone likely share mechanisims, however postural tone has a greater active component that prevents postural collapse. Note that the fraction between passive and active muscle tone depicted is only illustratve and depends on the specific muscle and postural context. **(B)** The involuntary tonic muscle activity during stance constitutes tone but holding a fist does not, because of the voluntary origin of the tonic contraction.

Two terms are used to refer to muscle tone: resting tone and postural tone, depending on the postural state of the subject. Both forms likely share passive and active mechanisms, although to different extents, and likely share neural circuitry (Figure 1A). Resting muscle tone refers to the tension when subjects are relaxed and fully supported; it is often assessed by passive resistance to movement (Foster and Rivers, 1892). Resting tension acts to prevent muscle slackness, although its function remains somewhat obscure. In the extremities, resting muscle tone is generally electrically silent (Masi and Hannon, 2008) but axial and proximal muscles are often active, even when subjects are supported and relaxed (Burke and Gandevia, 1993; Mense and Masi, 2010).

In contrast, postural muscle tone emphasizes the functional role of holding different parts of the skeleton in relation to each other (Gurfinkel, 2009); it is typically assessed during antigravity postural maintenance, where muscle activation is required to prevent collapse. Postural tone is typically < ~7% of maximal contraction (Gurfinkel et al., 2006) and is modulated by loading and by the mechanical demands to stabilize body posture. For instance, tonic activation of upper and lower body musculature is larger when standing than when lying down (Viir et al., 2007; Rubini et al., 2012), and it can disappear with the addition of external support (Masani et al., 2013). While the differences between resting and postural tone aren’t well understood, the main distinction may relate to the state of the subject during measurement. For instance, postural tone may involve increased activity in the same circuitry as resting tone. It is also possible that postural tone relies on somewhat different underlying circuitry.

### 2.2 Involuntary nature of muscle tone

Muscle tone is involuntary. It is behaviorally distinct from and engages different brain pathways than voluntary control (Guo et al., 2015; Teng et al., 2017; St George et al., 2018; Russell et al., 2022). Thus, the tonic leg extensor activation in stance constitutes tone but the sustained flexor activation when making a fist does not (Figure 1B). Muscle tone develops according to a genetically determined timescale, changing from flexor to extensor biased in early infancy, and sequentially developing postural support for the head and then body (Amiel-Tison, 2002; Lefèvre, 2002; Goo et al., 2018).

### 2.3 Measuring muscle tone

Various measures have been used to quantify muscle tone – all of which relate to muscle tension (Table 1). As it is difficult to noninvasively measure the series tension of a muscle, tone is often assessed by the resistance to passive joint motion though the change in force per unit length (Worman et al., 2023). This measure reflects the tensional changes in all muscles that cross the deformed region, including both lengthened and shortened muscles. However, mechanical measures do not identify the relative contributions of active vs. passive sources (Morin, 2016), which can be discriminated by EMG.

**Table 1:** Common measures of muscle tone.

Research assessment	Clinical assessment
Change in force per length	Passive resistance
Muscle hardness	Joint restriction
Myotonomotry	Pendulum test
Resonance frequency	Traction test
EMG	Abnormal posturing
	Clinical scales

Basic research and clinical measures all relate to muscle tension. EMG is typically used to identify active contributions.

Muscle tone can be inferred through muscle hardness (Fischer, 1987) or myotonometry, that quantifies the dynamic response to muscle belly deformation (Hu et al., 2018). Another indirect measure of tone is the resonance frequency of body segments, assessed with an oscillating perturbation (Walsh, 1992). While tone can also be estimated from postural alignment (Worman et al., 2023), this is region dependent and problematic due to muscular redundancy and anatomical differences.

Clinically, muscle tone can be assessed through the resistance to passive movement, joint restrictions, the pendulum test (Wartenberg, 1951), traction test (Jabbour and Clark, 1965), abnormal posturing, and clinical scales (Amiel-Tison, 2002; Reinhold and West, 2014; Goo et al., 2018).

Difficulty characterizing this distributed, adaptive, low-level system has hindered the understanding of muscle tone and its relation to other phenomena, such as performance and pain. Many measures of tone are indirect, and most do not discriminate between active and passive contributions (Worman et al., 2023). The small magnitude, constant nature, and contribution of deep muscles to tone often render surface EMG unreliable (Van Houtte et al., 2013). Characterizing muscle tone also requires evaluating the dynamic adaptive responses to yield and resist as well as cross-body interactions. There are many ways tone can be distributed, adaptive and interconnected, which make it impractical to comprehensively assess with a fixed protocol.

## 3 History of muscle tone research

The study of muscle tone has a long and complex history. Early research in the late 19th to mid-20th century focused on pathological animal models in order to exaggerate tone and eliminate voluntary activity, as active contributions to muscle tone are abolished by anaesthesia (Gurfinkel, 2009; Buchmann et al., 2014; Ivanenko and Gurfinkel, 2018). While nuanced observations from these animal models yielded insight into processes that regulate tone (e.g., Sherrington, 1915; Magnus, 1925; Rademaker, 1931), this was complicated by the induced pathology, which confounded the relevance to healthy subjects (Creed et al., 1932; Davidoff, 1992). Early experiments revealed various phenomena including neck effects (Magnus, 1925), lengthening and shortening reactions (Sherrington, 1909), and “magnetic reactions” (Rademaker, 1931).

However, the reflex nature of muscle tone that occurs in some decerebrate preparations led to an overgeneralisation in the literature, such that a reflex basis was assumed for all tone (Davidoff, 1992; Walsh, 1992; Mense and Masi, 2010; Ivanenko and Gurfinkel, 2018). Thus, the myotatic stretch reflex (Sherrington and Liddell, 1924) became erroneously accepted as the basis for healthy muscle tone. In some fields, muscle tone became defined narrowly as velocity dependent resistance to stretch (Lance, 1980). This definition was based on spasticity, which is now known to result from central factors, including decreased descending supraspinal drive, decreased synaptic inhibition, and increased intrinsic motoneuron excitability (Bennett et al., 2004; Zelenin et al., 2016; Thaweerattanasinp et al., 2020; Mahrous et al., 2024), rather than excessive peripheral feedback loop gain. In general, the central regulation of tone has received far less attention than peripheral contributions, particularly in neurologically healthy subjects, even though prominent models of motor control like the equilibrium point hypothesis (Feldman, 1966; Feldman, 1986) have highlighted central influences on stretch reflex thresholds for decades.

Simplistic reflex models, and difficulty defining and measuring muscle tone, contributed to the near abandonment of tone research in the latter 20th century, despite being poorly understood. However, an understanding of muscle tone is a clinical necessity, as neurological disorders commonly cause consequential disturbances to tone. Knowledge of healthy physiology provides context that is advantageous for understanding disorder. Unfortunately, due to the historical focus on pathological models, there has been a lack of research into the physiology of healthy muscle tone.

## 4 Properties of muscle tone

### 4.1 Distribution

Muscle tone is broadly distributed throughout the musculature. Because of mechanical redundancy, a given body configuration can be stabilized by many different distributions of activity (Latash, 2012). For instance, numerous combinations of superficial, deep, medial, lateral, and antagonistic muscles can be engaged to support the trunk. For this reason, body alignment alone does not reveal how tone is distributed across the muscles.

### 4.2 Adaptation

Muscle tone adapts dynamically to meet biomechanical requirements, varying across different postures to counteract changing static loads (O'Sullivan et al., 2006; Caneiro et al., 2010). While one maintains a posture, tonic activity increases to resist deformation to external loading (Marigold et al., 2004; van Drunen et al., 2013). The tone system is extremely sensitive to changes in position and force, allowing it to readily compensate for disturbances. Changes occur at extremely low displacements (<1 deg) and velocities (< 1 deg./s) (Gurfinkel et al., 1995a,b; Gurfinkel et al., 2006).

Muscle tone also adapts in the opposite way – to yield to a load, allowing posture to change, for instance when a barber turns your head, or a dance partner lifts your hand. This creates postural plasticity through “lengthening and shortening” reactions, which decrease tone during muscle lengthening and increase it during shortening (Sherrington, 1909; Walsh, 1992; Gurfinkel et al., 2006). Functionally, this allows muscles to change length while maintaining constant tension. Such compliant tonic reactions are prominent in healthy infants in the first year of life, and thus constitute an innate aspect of tonic control (Solopova et al., 2019; Dolinskaya et al., 2023).

### 4.3 Habitual and individual nature

While muscle tone can readily adapt to changing postural demands, it is also conservative. Muscle tone varies widely across people; several-fold differences in tone are commonly observed (Gurfinkel et al., 2006). Both the distribution of tone and its adaptivity at different body regions are highly individual. This manifests as marked individual preferences in tone and postural alignment that are stable over long time periods (Lestienne and Gurfinkel, 1988; Lacquaniti and Maioli, 1994; Gurfinkel et al., 2006; Cacciatore et al., 2011a; Wainio-Theberge and Armony, 2024). There is a tendency to drift back to one’s habitual posture over time (Duarte and Zatsiorsky, 2002), which presumably creates a consistent, stable platform for coordinating action.

### 4.4 Non-local influences

Muscle tone is influenced by both neighboring and distant parts of the body, such as neck position affecting limb tone (Gurfinkel and Levick, 1991; Gurfinkel et al., 1995a,b; Bruijn et al., 2013). Many non-local influences on tone have been observed, including from the jaw to the neck and trunk (Lippold et al., 2006; Giannakopoulos et al., 2013; Julia-Sanchez et al., 2019), from the trunk to the neck and eyes (Bexander and Hodges, 2023), and from the limbs to the trunk (Kluzik et al., 2007). Interactions also occur within (Gurfinkel et al., 1989; Solopova et al., 2019; Dolinskaya et al., 2023) and across limbs (Zelenin et al., 2015; Solopova et al., 2019; Dolinskaya et al., 2023). In addition, haptic finger contact has been observed to affect hip tone (Franzén et al., 2011). Cross-body interactions can result from the mechanics of interconnected kinematic or myofacial chains (Loram et al., 2016; Wilke et al., 2016; Kellis et al., 2024) or can be mediatiated by neural circuits (Zelenin et al., 2015; Giannakopoulos et al., 2018).

## 5 Neurophysiology of muscle tone

**Figure 2:**
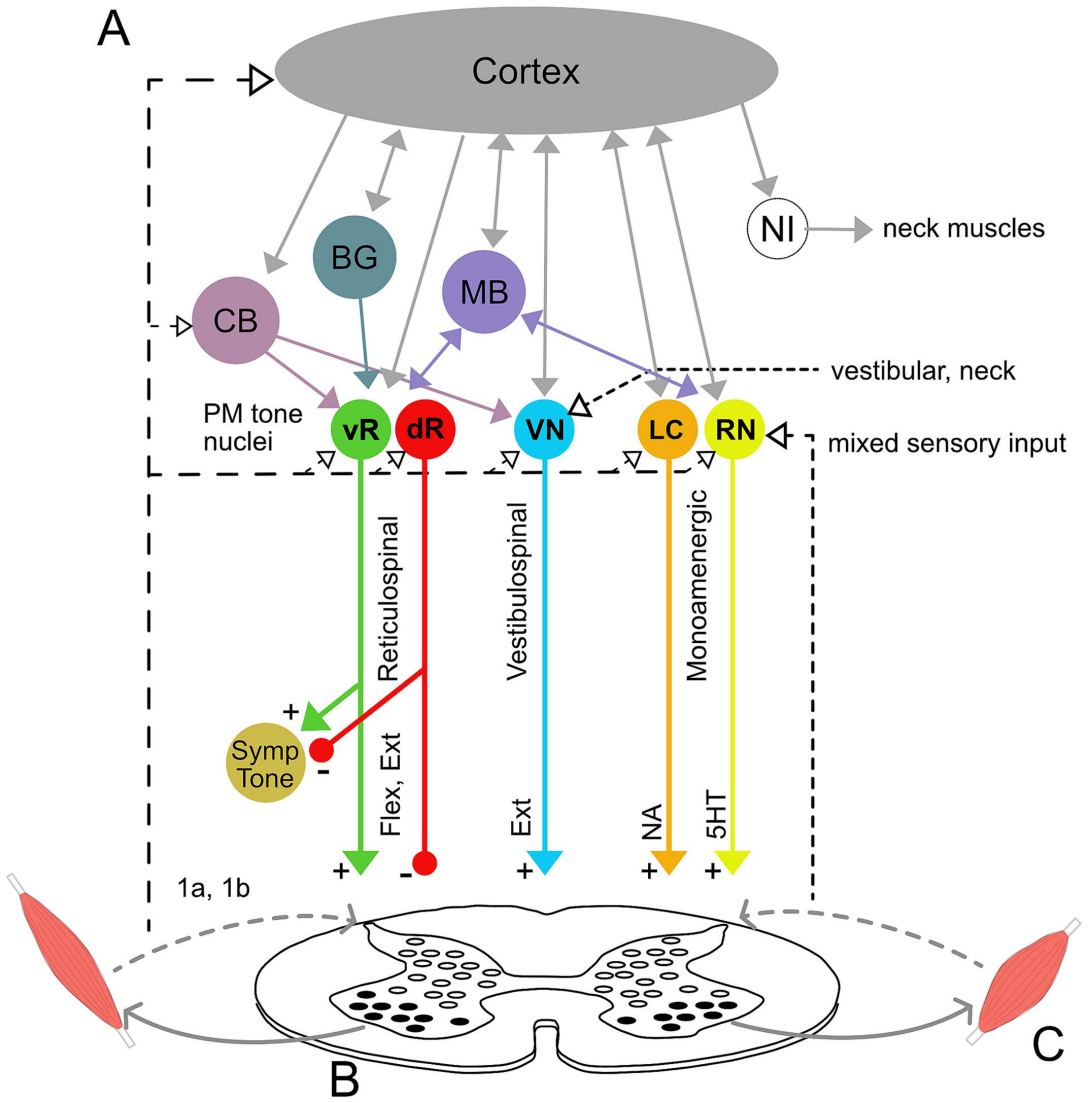
Neural circuitry underlying muscle tone. **(A)** Descending brainstem and supraspinal circuitry. Pontomedullary nuclei (PM tone nuclei) provide parallel descending tonic drive to the spinal cord to activate and modulate the excitability of spinal circuits through reticulospinal, vestibulospinal, and monoaminergic tracts. Solid and dashed lines indicate central and afferent projections, respectively. Pontomedullary tone nuclei include tone-excitatory regions in the ventral medullary reticular formation (vR), tone-inhibitory regions in the dorsal medullary reticular formation (dR), excitatory projections from the vestibular nuclei (VN), and two groups of monoaminergic nuclei: the noradrenergic locus coeruleus (LC), and serotonergic raphe nuclei (RN). The raphe nuclei are subdivided into caudal nuclei, which send descending projections to the spinal cord, and rostral raphe nuclei, which send ascending projections to the forebrain. Descending projections from the vR and dR also branch to co-regulate sympathetic nervous system activity. The monoaminergic tracts release noradrenaline (NA) and serotonin (5HT) onto motoneurons and diffusely across the spinal cord. The pontomedullary tone nuclei receive inputs from higher level structures, including the cerebral cortex, basal ganglia (BG), cerebellum (CB) and midbrain (MB), which includes the pedunculopontine nucleus, cuneiform nucleus, and periaqueductal gray. The neural integrators for the neck (NI) are also located in the midbrain and project to neck motoneurons. Note that known interconnections, for example those between the pontomedullary nuclei, are omitted for clarity. **(B)** Feedback circuitry in the spinal cord. Spinal cord feedback circuits are capable of adapting tone to postural demands; however, they require tonic excitation to function. These circuits consist of heterogeneous populations of interneurons (white ovals) distributed across spinal cord laminae. These interneurons differentially receive input from 1a stretch receptors and 1b tendon organs from ipsi-and contralateral limbs, respond to different stimuli, and project to motoneurons (black ovals). **(C)** Flexor and extensor muscles. The reticulospinal and monoamine tracts project to both flexors and extensors, while the vestibulospinal tract projects solely to extensors. Note that while only a pair of muscles are shown, muscle tone is broadly distributed across the musculature and thus involves activation throughout the spinal cord.

Current understanding of muscle tone neurophysiology comes largely from animal models. Both central pathways and peripheral feedback are involved (Figure 2). Central pathways from the mesopontine and pontomedullary reticular formation regulate tone through parallel descending excitatory and inhibitory projections (Takakusaki et al., 2016). Stimulating these regions directly enhances and suppresses tone, respectively (Magoun and Rhines, 1946; Mori et al., 1982; Mori, 1987; Musienko et al., 2008). Peripheral pathways adapt tone to the environment through parallel feedback loops within the spinal cord, brainstem and supraspinal structures such as the cerebellum (Matsuyama and Drew, 2000a; Deliagina et al., 2014; McCall et al., 2017).

Muscle tone is also influenced centrally through neuromodulator systems: serotoninergic projections from the raphe nuclei and noradrenergic projections from the locus coeruleus increase tone (Kiyashchenko et al., 2001; Jacobs et al., 2002; Takakusaki et al., 2016). Cholinergic projections within the brainstem decrease muscle tone and are involved in sleep atonia (Nishino et al., 1995; Takakusaki et al., 2016).

### 5.1 Spinal networks

Much of the feedback circuitry that regulates muscle tone is located within the spinal cord. These circuits have been characterized using tilts of the support surface in quadrupeds (for review see Deliagina et al., 2014), which results in phasic and tonic changes in muscle activity to stabilize the body. The underlying spinal cord circuits are complex, being comprised of hundreds of heterogeneous interneurons (Figure 2B) that are distributed across the spinal cord laminae and that act in different phases of tilt (Zelenin et al., 2015). These spinal circuits form distinct front-and hind-limb feedback controllers that integrate afferent information from ipsi-and contra-lateral limbs to adapt tone to different planes of surface tilt. This architecture differs fundamentally from the monosynaptic stretch reflex, to which tone is often misattributed (Davidoff, 1992; Masi and Hannon, 2008; Mense and Masi, 2010). Resistive and compliant tonic reactions also likely involve oligosynaptic feedback pathways within the spinal cord and possibly also the brainstem (Solopova et al., 2019; Dolinskaya et al., 2023).

### 5.2 Descending supraspinal tonic drive

Although feedback circuits are contained within the spinal cord, tonic excitation is essential for them to function (Deliagina et al., 2014). This tonic drive is usually provided by vestibulospinal, reticulospinal, and monoaminergic descending input (see Figure 2) and acts to provide an excitability bias that “switches on” the spinal circuits. Interrupting communication between the brainstem and spinal cord abolishes muscle tone and postural responses (Zelenin et al., 2013). However, various forms of excitatory input, such as constant epidural stimulation, vibration, or strong cutaneous input, can substitute for the intrinsic descending drive and activate spinal feedback circuits, even in the isolated spinal cord (Musienko et al., 2010; Lavrov et al., 2015; Takakusaki et al., 2016). Thus, descending systems provide an essential but unspecific tonic input that increases the excitability of spinal networks (Musienko et al., 2008; Deliagina et al., 2014).

Tonic drive may serve as an important mechanism for regulating muscle tone by modulating spinal excitability (Gurfinkel et al., 1999). The importance of excitability for muscle tone regulation is clear from many observations, including: changes in tonic muscle activity following vibration and post-contraction (Gurfinkel et al., 1989; Gurfinkel et al., 1995a; Gurfinkel et al., 1999; Gurfinkel et al., 2006); fluctuations in state following decerebration (Sherrington, 1909; Beritoff, 1915; Musienko et al., 2008; Zelenin et al., 2013); the influence of remote voluntary effort on tone (Walshe, 1923; Wolff et al., 1983; Yanagisawa and Hashimoto, 1995; Jiang et al., 2021); and excitability-related facilitation of lengthening and shortening reactions (Gurfinkel et al., 2006; Solopova et al., 2019; Dolinskaya et al., 2023). Tonic drive may act to switch on spinal circuits (Musienko et al., 2008; Ivanenko et al., 2017), set response levels (Musienko et al., 2008), and specify whether to resist or yield to loading (Gurfinkel et al., 1989; Gurfinkel et al., 2006). This is reminiscent of Bernstein’s (1940) statement that, “Tonus as an ongoing physiological adaptation and organization of the periphery is *not a condition of elasticity but a condition of readiness*.”

#### 5.2.1 Vestibulospinal pathways

The various descending pathways affect posture in different ways. Tonic vestibulospinal drive reflects the position and motion of the head and projects predominantly to extensor muscles, providing a relatively constant drive that facilitates antigravity support (Matsuyama and Drew, 2000a; McCall et al., 2017). Disruption of the vestibulospinal drive decreases extensor tone and causes postural instability (Orlovsky, 1972; Matsuyama and Drew, 2000a). This pathway also mediates interactions between the head and body, for instance head position affecting limb tone (Bruijn et al., 2013; Beraneck et al., 2014). Functionally, modulating vestibular drive between left and right sides alters limb posture by tonically biasing the set points of spinal circuits (Hsu et al., 2012).

#### 5.2.2 Reticulospinal pathways

Reticulospinal neurons are generally involved in posture and movement, with diverse and heterogenous effects (McCall et al., 2017). Like vestibulospinal pathways, reticulospinal neurons mainly terminate on spinal interneurons (McCall et al., 2017; Zhang et al., 2024) and have much larger branching patterns than corticospinal projections (Baker, 2011). The tone excitatory and inhibitory systems (Takakusaki et al., 2016) form part of the reticulospinal projection, which is generally modulated by postural demands (Matsuyama and Drew, 2000b; Schepens et al., 2008; Russell et al., 2022). Neurons in the ventral pontomedullary reticular formation act to increase tone, while more dorsally located neurons decrease tone (Takakusaki et al., 2016). Many reticulospinal neurons are also active during movement (Matsuyama and Drew, 2000b; Schepens et al., 2008). While some reticulospinal neurons respond generally to perturbations and could provide unspecific excitation to the spinal cord, others respond more selectively and may play a more specific role in coordination, for example triggering particular postural responses (Matsuyama and Drew, 2000b; Stapley and Drew, 2009; Musienko et al., 2014; Brownstone and Chopek, 2018), coordinating posture with movement (Schepens et al., 2008), or acting as part of supraspinal feedback loops that modulate tone. Reticulospinal muscle tone pathways also project to the autonomic nervous system and co-regulate sympathetic tone (Zhang et al., 2024).

#### 5.2.3 Monoaminergic pathways

Descending monoaminergic projections affect the overall sensitivity of the motor system. This occurs in part through dramatically affecting the input–output gain of motoneuron pools (Heckman et al., 2003; Wei et al., 2014), thereby affecting muscular recruitment. While monoaminergic systems project diffusely throughout the spinal cord and brain, projections to axial regions are denser than projections to distal regions (Jacobs et al., 2002). Monoaminergic modulation acts more slowly than ionotropic synaptic transmission and can exert effects lasting from seconds to minutes. This produces sustained firing in fatigue-resistant motor units, particularly important for posture, via persistent inward currents that cause a prolonged excitatory state (Heckman et al., 2003).

Both noradrenergic and serotonergic drive increase muscle tone (Kiyashchenko et al., 2001; Jacobs et al., 2002; Musienko et al., 2008). Noradrenergic drive increases with vigilance and arousal (Aston-Jones et al., 2001) while serotonergic drive reflects overall motor demand (Jacobs et al., 2002). Descending serotonergic modulation from caudal raphe nuclei underlies the tonic vibration reflex, considered to be a model of postural tone (Gurfinkel et al., 1999; Wei et al., 2014).

### 5.3 Higher level brain structures

Because muscle tone is present after decerebration, it is predominately generated in the hindbrain and spinal cord. However, it is influenced by other brain structures, such as the cerebellum, basal ganglia, limbic system and cortex (Beloozerova et al., 2003a; Musienko et al., 2008).

#### 5.3.1 Cerebellum and basal ganglia

The cerebellum facilitates muscle tone (Asanome et al., 1998); midline and lateral cerebellar damage causes hypotonia of the body axis and limbs, respectively (Bodensteiner, 2008). The basal ganglia also alter tone (Martin, 1967; Chastan et al., 2009), and basal ganglia dysfunction can underlie a range of pathologies including rigidity and cervical dystonia. Both the cerebellum and basal ganglia may act through projections to tone-regulating mesopontine tegmental nuclei (Asanome et al., 1998; Takakusaki et al., 2004).

#### 5.3.2 Limbic system

Limbic regions mediate emotion-related changes in tone via the brainstem (Holstege et al., 1996; Nishino, 2003). Stress generally increases tonic muscle activity (Eijckelhof et al., 2013). Notably, stress-increased muscle tension is mediated by descending reticulospinal connections (Marker et al., 2017) and is not affected by blocking the sympathetic nervous system (Nilsen et al., 2008). Limbic regions also presumably underlie the reported correlation between habitual tone and personality traits (Wainio-Theberge and Armony, 2024).

#### 5.3.3 Cerebral cortex

Lesions to the cerebral cortex can cause spasticity and flexor-biased postures (Chang, 2005), suggesting the cortex can also influence muscle tone in absence of neurological damage. Moreover, neurons in primary motor cortex are modulated with static lateral tilts of the support surface (Beloozerova et al., 2003b; Beloozerova et al., 2005; Karayannidou et al., 2009). This modulation occurs via proprioceptive limb afferents (Deliagina et al., 2008), supporting cortical involvement in the feedback modulation of tone. However, some studies have reported that cortical motor neurons tend to only fire transiently during sustained postures (Shalit et al., 2012). That postural tone develops before the cortex produces functional output (Blumberg and Adolph, 2023) and persists during global cortical inhibition (Guo et al., 2015) suggests that the cerebral cortex is not the main locus of tone regulation. However, it is likely important for mediating cognitive and attentional changes to tone via extrapyramidal pathways through the brainstem.

The cortex exhibits sensorimotor beta rhythms during periods of sustained posture (Tia and Pifferi, 2021; Easthope et al., 2023). While the role of the beta rhythm has been controversial (Kilavik et al., 2013), it acts as a form of gain modulation (van Elswijk et al., 2010) different from slow acting monoamine systems, and it may relate to volitional postural maintenance.

### 5.4 Afferent input

Muscle tone is influenced by a heterogeneous assortment of afferent input. This sensory influence occurs through more than just simple feedback loops, but via a complex integration of sensory inputs suggestive of a “body scheme” (Gurfinkel and Levick, 1991; Gurfinkel et al., 1995a,b; Kluzik et al., 2007; Franzén et al., 2011). At the spinal level, feedback circuits receive input from 1a and 1b afferents (Deliagina et al., 2000), which both facilitate muscle tone (Pratt, 1995; Duysens et al., 2000; Van Doornik et al., 2011). Thus, spinal feedback circuits respond to information reflecting muscle state.

Sensory input also acts supraspinally, for instance through vestibular organs that affect vestibulospinal drive. The descending monoaminergic pathways respond to a variety of sensory stimulation, including vibration, cutaneous input, sound, and particularly deep pressure (Moolenaar et al., 1976; Lapole et al., 2023). This non-specific sensitivity of descending tonic drive could underlie the diversity of ways that afferent inputs, ranging from light touch to deep massage, can influence tone.

#### 5.4.1 Joint position affects excitability

Spinal excitability is also influenced by joint position (Hyngstrom et al., 2007; Nuzzo et al., 2016). Muscle stretch increases the excitably of agonists via 1a afferents and decreases antagonist excitability through reciprocal inhibition (Johnson and Heckman, 2014). However, joint position can also affect excitability in remote regions. For instance, shoulder position affects the excitably of hand muscles (Geed et al., 2021) and whole body posture affects the excitably of arm muscles (Runnalls et al., 2017).

### 5.5 Postural setpoints

Perhaps surprisingly, it is not clear how habitual postural preferences are represented in the nervous system. While postural setpoints are affected by descending vestibulospinal drive (Hsu et al., 2012), this signal reflects “operative” changes that modulate setpoints according to current demands (Lestienne and Gurfinkel, 1988), as opposed to habitual postural preferences. Postural setpoints are also influenced by neural integrators (Shadmehr, 2017; Albert et al., 2020), which set postural tone based on the preceding movement command. These might underlie how “posture follows movement like a shadow” (Sherrington, 1906) as well as forms of cervical dystonia (Shaikh et al., 2016). Neural integrators have been identified for the eye and neck in the midbrain (Shadmehr, 2017) and have been demonstrated to exist for the limbs (Albert et al., 2020), but their anatomical location is not yet known. However, neural integrators are also likely operative, as they set tone levels based on recent movement. It is possible that long-term habitual setpoints are manifestations of the brainstem and associated descending tonic drives (Thiele et al., 2014), however they could also be encoded at higher levels, such as the body scheme (Lestienne and Gurfinkel, 1988; Romano et al., 2021). Another possibility is that habitual postures are stored in a distributed fashion, through long-term stable properties across multiple levels of the nervous system.

### 5.6 Relations among tone, voluntary movement, and anticipatory postural adjustments

Evidence from multiple sources suggests that tone and voluntary movement constitute distinct forms of behavior. (1) Cortical inhibition suppresses voluntary reaching but does not affect muscle tone (Guo et al., 2015). (2) Axial tone is impaired in idiopathic camptocormia in the absence of attention, but the trunk can be held upright voluntarily (St George et al., 2018). (3) Muscle tone and movement commands are adapted independently via separate cerebellar loops (Shadmehr, 2017). (4) Postural tone and voluntary contraction differentially activate brainstem and corticospinal tracts, respectively (Teng et al., 2017; Russell et al., 2022).

Despite the behavioral distinction between muscle tone and movement, they share some overlapping circuitry. For instance, neurons in the brainstem participate in both movement and tone regulation (Schepens and Drew, 2004; Schepens et al., 2008; Gibson et al., 2023), and descending monoaminergic gain control pathways affect muscle recruitment during voluntary actions (Heckman et al., 2003; Wei et al., 2014). In addition, gait coordination is affected by vestibulospinal projections that also influence tone (Gottschall and Nichols, 2007; Gottschall and Nichols, 2011).

The Gurfinkel et al.’s (1999) “Lock with Two Keys” framework provides a conceptual basis for the involvement of both tonic and phasic commands in movement. Tonic commands set excitability levels to prepare lower circuits for action while phasic commands initiate and coordinate the movement. Consistent with this view, tonic drive from the various descending brainstem projections configures the spinal cord, while outputs from cortical motor and premotor regions and central pattern generators coordinate the phasic mechanisms. Evidence supporting the role of tonic commands in movement control includes findings that increasing excitability of spinal circuits can invoke gait rhythms (Solopova et al., 2015; Ivanenko et al., 2017).

Movement is also associated with anticipatory postural adjustments (APAs), which provide targeted stabilization for predictable movement-related disturbances before movement onset. However, it is currently unclear whether APAs involve changes to muscle tone. As tone represents ongoing activity, it is difficult to determine whether movement-related changes result from anticipatory feedforward processes or from sensory feedback that occurs later as a result of the movement. Evidence for the idea that anticipation affects muscle tone includes findings that reticulospinal neurons are activated before voluntary limb movement (Schepens and Drew, 2004; Schepens et al., 2008), mechanical disturbance is decreased to changes in sustained postural load when subjects trigger the unloading themselves (Viallet et al., 1992), and that humans adjust their neck posture in preparation for walking (Baer et al., 2019). Evidence against the idea that APAs affect muscle tone include findings that predictable platform tilts do not cause anticipatory changes in tone (Beloozerova et al., 2005) and observations that APAs tend to be phasic (Belen'kii et al., 1967; Cordo and Nashner, 1982; Viallet et al., 1992; McIlroy and Maki, 1999; Caronni and Cavallari, 2009; Lee et al., 2009; Cohen et al., 2017). For example, tapping a finger induces transient anticipatory changes in arm and back muscles to counteract movement-related interaction torques (Caronni and Cavallari, 2009), which are too brief to represent muscle tone. One way that postural tone does “anticipate” movement is through a general increase in tone beforehand, for example prior to gait initiation, which provides general mechanical stiffness that resists movement related perturbations (Mori, 1987).

## 6 Muscle tone, performance, and pain

### 6.1 Muscle tone and movement

Muscle tone can affect movement. For instance, the flexed posture present in newborns prevents locomotion, even in the presence of weight support, until extensor tone is predominant (Amiel-Tison, 2002; Wilson et al., 2022). Movement coordination is degraded with tone pathologies; both hyper-and hypotonia alter sit-to-stand coordination (Ha and Sung, 2023) and hinder overall motor performance (Goo et al., 2018). Muscle tone is necessary for locomotion and closely integrated with the underlying neural circuitry. Stimulating excitatory and inhibitory brainstem tone regions can initiate and terminate gait, respectively (Mori, 1987; Takakusaki et al., 2016).

Axial muscle tone appears to be particularly important for motor function. Atypical axial tone is a major sign of disease or disability in infancy and its evaluation features centrally in early neurological assessments (Haataja et al., 1999; Amiel-Tison, 2002; Leyenaar et al., 2005; Romeo et al., 2016). Axial tone also affects gait coordination (Martin, 1967; Ivanenko and Gurfinkel, 2018) and is correlated with rolling and turning impairment in Parkinson’s disease (Franzen et al., 2009). In addition, providing head support to newborns results in the expression of movements that are typically seen in much older infants (Amiel-Tison and Grenier, 1983), and providing trunk support to six-month-old infants leads to much smoother reaching movements (Hopkins and Rönnqvist, 2002). The importance of axial tone may stem from its essential role in stabilizing the spine (Lucas and Bresler, 1960). Moreover, the complexity and redundancy of axial musculature allows many possible distributions, including ones that may interfere with movement, for example through altered mechanical interactions between limbs (Gurfinkel et al., 2006). However, axial tone may be primarily important for neurological reasons. For instance, poor axial tone could degrade coordination by disrupting muscular recruitment.

In neurologically healthy subjects, muscle tone affects various movements including scapular kinematics in swimmers (Chen et al., 2023), speech production after spaceflight (Shamei et al., 2023), and vocal coordination in vocal tension dysphonia (Hocevar-Boltezar et al., 1998; Kooijman et al., 2005; Tomlinson and Archer, 2015). Alterations to muscle tone can also affect pelvic function (Worman et al., 2023) and respiration (Austin and Ausubel, 1992). Tone may also affect the smoothness of whole body movements (Cacciatore et al., 2014). The general effects of tone on movement coordination suggest that muscle tone may be an important determinant of motor performance, consistent with Bernstein’s theory that tone forms the foundational level of the motor system (Latash et al., 1996; Profeta and Turvey, 2018).

#### 6.1.1 Mechanisms underlying tone’s influence on movement

**Figure 3:**
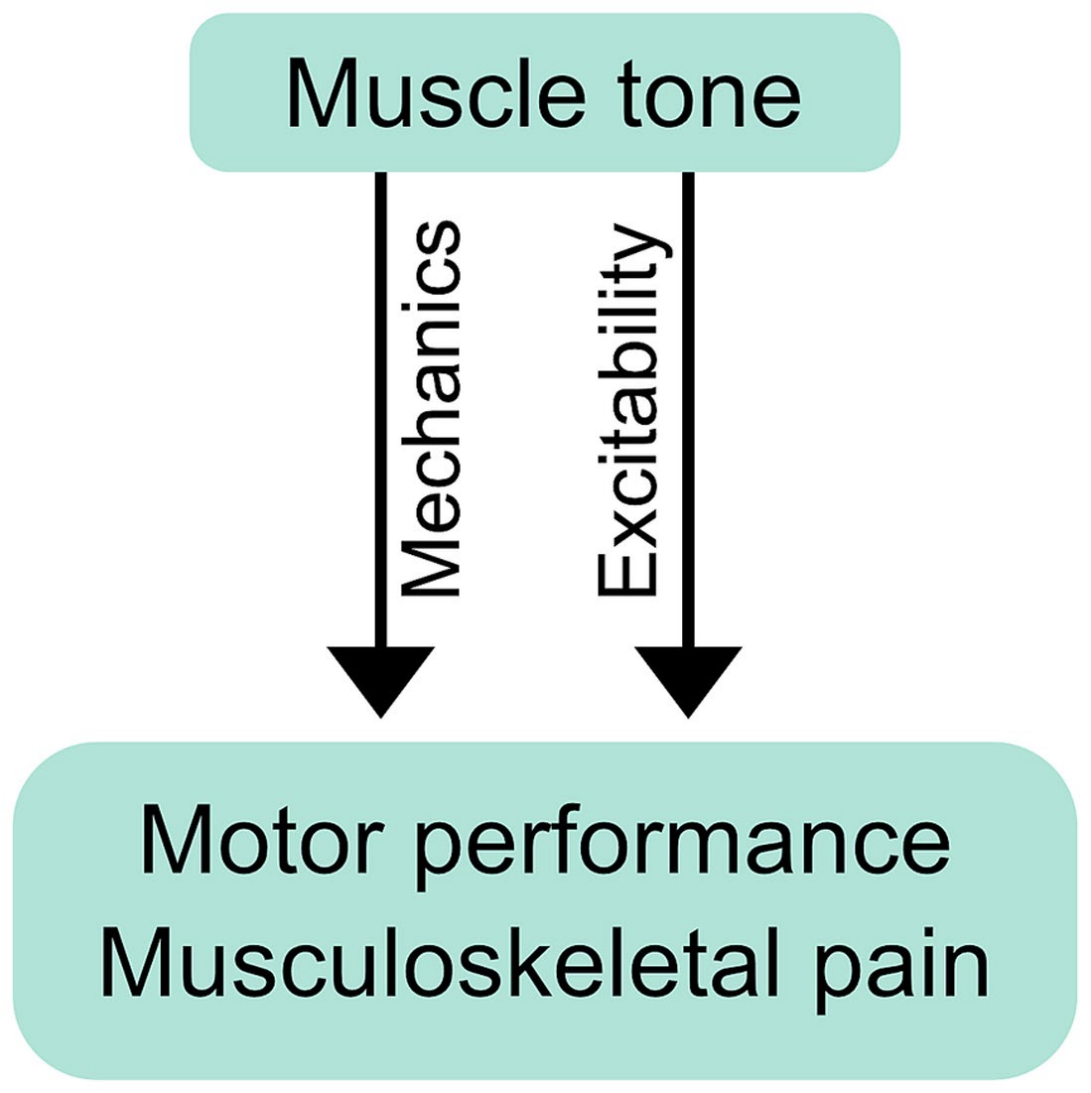
Mechanical and neurological influence of muscle tone on performance and pain. Postural tone creates a distributed network of stiffness and resistance (i.e., “postural frame”) that can mechanically affect movement coordination and musculoskeletal pain. Tone can also exert its influence neurologically, through changes in excitability, which may influence performance and pain through the resulting changes in neural responses.

Muscle tone may affect movement through mechanical or neurological mechanisms (Figure 3). Mechanical effects result from how the resistive and compliant behavior of the body affects movement. The properties of postural tone that stabilize body configuration also create a distributed network of stiffness and resistance (i.e., “postural frame”), which affects range of motion (Wendt and Waszak, 2024) and may shape the coordination of whole body movements such as sit-to-stand (Cacciatore et al., 2014). We consider this a mechanical influence as the effects on movement occur through resistance and compliance.

Muscle tone can also influence movement neurologically, through the excitability of neural circuits. Descending tonic drive alters the excitability of motoneurons, which can affect muscle recruitment during voluntary action (Stokes and Young, 1984; Kavanagh and Taylor, 2022). Examples include abnormal muscle recruitment during voluntary movement in people with hypertonia (Ha and Sung, 2023) and excessive neck tension (Redenbaugh and Reich, 1989; Hocevar-Boltezar et al., 1998). Excitability can influence movement more broadly by facilitating any or all of: tonic reactions that affect resistance and compliance (Gurfinkel et al., 2006; Musienko et al., 2010), cross-body interactions (Gurfinkel et al., 1995a), and circuit primitives that underlie movement coordination (Ivanenko et al., 2017). Excitability can also bias movement, for example causing subjects to walk along a curved trajectory (Ivanenko et al., 2006).

#### 6.1.2 Muscle tone affects balance

Balance refers to the dynamic interplay of the whole body centre of mass and under-foot centre of pressure; it differs from static postural control and tone regulation (Ivanenko and Gurfinkel, 2018). Neck tone influences postural sway (Julia-Sanchez et al., 2019), ankle stiffness degrades balance (Warnica et al., 2014), and increased muscle tone can restrict balance strategies (Kaminishi et al., 2021). Interventions targeting muscle tension also improve balance (Nacci et al., 2012; Faralli et al., 2017), which supports the relevance of muscle tone to balance control.

### 6.2 Muscle tone and pain

While pain is multifaceted and complex, muscle tone and pain are interrelated. Altered tone has been reported for many chronic musculoskeletal pain conditions including neck (Falla et al., 2004), back (van Dieen et al., 2003), and pelvic pain (Worman et al., 2023). Changes associated with pain include increased muscular stiffness (Alcaraz-Clariana et al., 2021a; Alcaraz-Clariana et al., 2021b; Li et al., 2022; Kurashina et al., 2023; Proulx et al., 2023; Vatovec and Voglar, 2024) and altered distribution of tonic EMG (van Dieen et al., 2003; Dankaerts et al., 2006; Mork and Westgaard, 2006; Planken et al., 2010; Park et al., 2013; Claus et al., 2018). Further links between muscle tone and pain are implied by coincident changes in tone and pain following intervention (Wong et al., 2015; Buttagat et al., 2016). Notably, tone is altered during deep sleep in people with chronic neck pain (Mork and Westgaard, 2004; Aldabbas et al., 2024), reflecting the automatic and subcortical origin of disruptions in tone.

#### 6.2.1 Pain-related disruptions to tone

In general, pain-related motor changes are highly individual (Wernli et al., 2020); muscle tone can increase, decrease or remain unchanged with pain (Claus et al., 2018; Hodges and Danneels, 2019). Back pain is associated with greater extremes and variation in axial tone across subjects (Cacciatore et al., 2011a), possibly reflecting “tight” and “loose” control strategies (van Dieën et al., 2019). Alterations are muscle-dependent; superficial muscles are more likely to increase their activity while deeper muscles tend to become less active (Falla et al., 2004; Hodges and Danneels, 2019). The adaptability of postural tone may also be affected, as people with back pain modulate their tonic muscle activity less across different postures than pain-free subjects do (Shirado et al., 1995; Claus et al., 2018; Gouteron et al., 2022).

#### 6.2.2 Causality of muscle tone and pain

While disruptions to muscle tone may act to cause pain, they may alternatively result from pain. In extreme cases, such as pathologies of tone, it is clear that alterations can lead to pain. For instance, hypertonia can cause pain through abnormal loading and structural changes in joints (Ostojic et al., 2019). Subjects with Parkinson’s disease also commonly experience neck and back pain, which accompanies rigidity (Alwardat et al., 2019). At the other extreme, hypotonia often results in joint pain due to the lack of postural support (Miller, 2020).

Some evidence suggests that muscle tone may contribute to pain in the absence of neurological pathology. Recovery from low back pain was associated with subjective reports of suppleness and “relaxed posture and movement” as well as decreased back muscle EMG (Wernli et al., 2022). Similarly, pain decreased in subjects with muscle tension dysphonia following treatment aimed at reducing tension (Tomlinson and Archer, 2015). These pain reductions were coincident with increased mobility.

Of course, pain results from many factors besides muscle tone, including nociceptive, central and psychosocial drivers. Even if abnormalities in tonic muscle activation result from pain, for example through nociceptive pathways distinct from those that regulate tone in the absence of pain – tone regulation may still be clinically relevant. Tone circuitry is likely to be involved in long-term maladaptive changes that act to perpetuate pain (Hodges and Smeets, 2015), and therefore renormalising muscle tone may be generally beneficial to pain outcomes.

#### 6.2.3 Mechanisms for influencing pain

##### 6.2.3.1 Tissue loading

Muscle tone might affect pain through tissue loading (Hodges and Danneels, 2019). While extreme changes in loading occur with tone pathology, more subtle changes may exacerbate musculoskeletal pain in neurotypical subjects.

##### 6.2.3.2 Excitability

Muscle tone may influence pain through underlying changes in excitability. Altered excitability has been proposed as a general mechanism underlying a diversity of pain-related motor deficits, including changes in movement coordination and muscle tone (Hodges and Tucker, 2011). Excitability may also influence the experience and development of pain.

Excitability is altered across cortical and subcortical levels in people with pain. Corticospinal excitability is decreased in people with low back pain (Strutton et al., 2005; Tsao et al., 2008; Massé-Alarie et al., 2012; Chiou et al., 2014) and is associated with pain severity, chronicity and functional impairment (Flor et al., 1997; Tsao et al., 2008; Schabrun et al., 2017). Moreover, reduced excitability within the sensorimotor cortex predicts the transition from acute to chronic low back pain (Jenkins et al., 2022; Jenkins et al., 2023). However, changes in corticospinal excitability with back pain mainly reflect cortical plasticity (Chiou et al., 2016) and may not underlie alterations in muscle tone.

Changes in muscle tone are more likely to relate to altered excitability within the brainstem and spinal cord. For instance, altered spinal excitability underlies increased tone of painful myofascial trigger points (Buchmann et al., 2014). People with neck pain have increased gains of the tonic cervico-ocular reflex, which adapts gaze position to accommodate changes in neck angle (de Vries et al., 2016). This suggests that neck pain is associated with increased excitability within vestibular pathways. Acute pain causes “reflex inhibition,” decreasing muscular recruitment (Stokes and Young, 1984; Indahl et al., 1997) through reduced spinal excitability, which typically affects deep muscles (Hodges et al., 2009). Conversely, greater activation of superficial muscles in people with pain may result from increased excitability within respective motoneuron pools (Hodges and Danneels, 2019), which acts to project the injured region. While it is possible that excitability changes are due to global increases or decreases in tonic drive, altered nociceptive reflexes with chronic back pain suggest that alterations are more complex and reflect a modified organization of spinal networks (Massé-Alarie et al., 2023).

Interestingly, the monoaminergic pathways from the caudal raphe nuclei that regulate motor gain overlap with the descending pain modulation system that regulates nociceptive inflow (Wang and Yasumitsu, 1994; Musienko et al., 2008). This pathway is compromised in people with pain and is highly clinically relevant (Ossipov et al., 2014; Ganley et al., 2023). In particular, descending monoaminergic drive diffusely modulates spinal excitability, consistent with both reducing pain and readying the motor system for action. Thus, influencing muscle tone through this pathway may act to reduce pain by coincidentally decreasing nociceptive input.

## 7 Altering muscle tone

**Figure 4:**
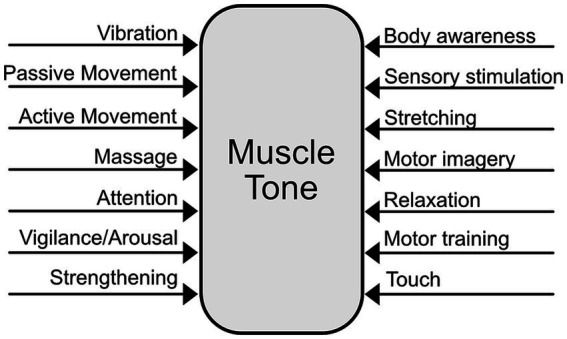
Multifactorial influences on muscle tone.

Because muscle tone is a distributed, interconnected, and automatic system, it is challenging to address clinically. The conservative nature of tone regulation may make it difficult to achieve beneficial long term changes. Moreover, altering tone in a way that positively affects pain may require addressing not only muscle tension, but also underlying states of excitability. In practice, many modalities are employed to alter muscle tone (Figure 4). Some of these, including vibration (Eklund and Hagbarth, 1966; Lavrov et al., 2015), massage (Sefton et al., 2011), sensory stimulation (Maher et al., 2013; Lavrov et al., 2015; Calamita et al., 2018), static motor imagery (Fairweather and Sidaway, 1993; Cohen et al., 2015) and training (Cacciatore et al., 2011a) have been reported to alter muscle tone.

**Figure 5:**
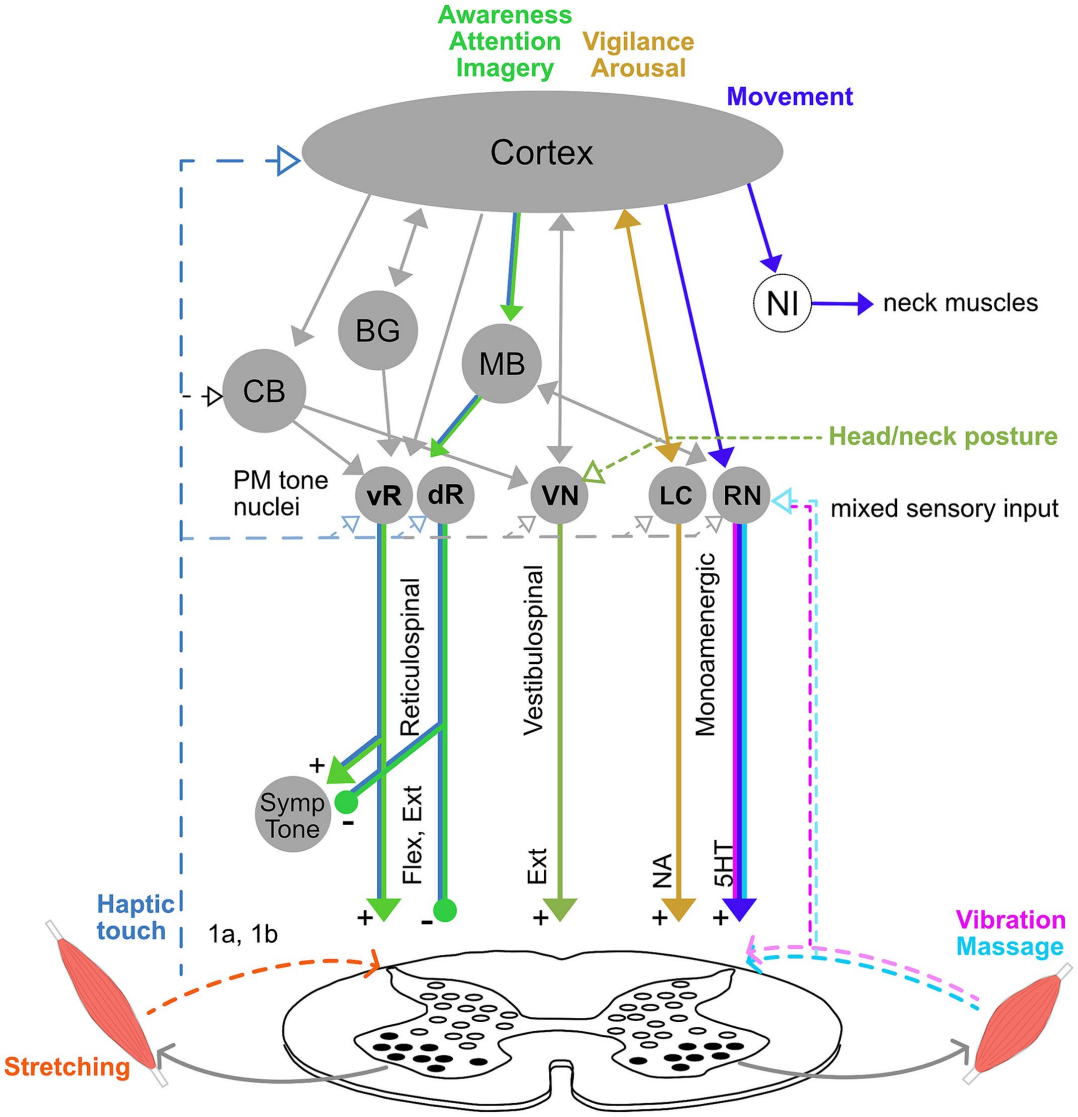
Model of intervention pathways for muscle tone. Tone can be influenced through a diverse combination of pathways, which likely underlie differences in the distribution, adaptivity, and interaction across the body. For instance, vibration changes tone through serotoninergic pathways from caudal raphe nuclei (RN), while attention and static motor imagery involve the cortex and likely also tonic reticulospinal pathways (vR, dR). Effects of vigilance and arousal on tone are mediated through the locus coeruleus (LC), and movement affects tone though RN, both of which influence overall motor gain. Movement also affects tone through neural integrators (NI), which set tone levels after a movement, and have been located for the neck in the midbrain. Head orientation and neck posture affect tone through the vestibular nuclei (VN). Haptic touch could influence tone via loops through the brainstem, cerebellum and cortex. In contrast, stretching is likely to alter tone locally though spinal cord circuitry. Note that this diagram is meant to illustrate the diversity of potential pathways and not to be exhaustive.

The multiple pathways involved in tone regulation suggest that muscle tone can be influenced in diverse ways (Figure 5). For instance, noradrenergic and serotonergic pathways influence tone globally thorough arousal and motor activity (Jacobs et al., 2002; Monjo and Shemmell, 2020). In contrast, reticulospinal pathways can cause more local changes in muscle tone and facilitate specific postural reactions (Schepens et al., 2008; Takakusaki et al., 2016; Lemieux and Bretzner, 2019). Vestibulospinal pathways carry tonic influences from the head and neck, which may be clinically important (Amiel-Tison and Grenier, 1983; Bruijn et al., 2013). Tone can be influenced from the bottom-up through peripheral sensory stimulation (Moolenaar et al., 1976; Lapole et al., 2023) and centrally via extensive cortical projections to brainstem descending pathways (Keizer and Kuypers, 1989).

### 7.1 Neglecting the underlying system

Despite the sophistication of tone regulation, it is often addressed simplistically, without reference to the nature of the underlying system. While muscle tone is dynamic, interconnected across the body, and closely related to excitability, it is often addressed locally through stretching and strengthening. Sometimes the main aim is only to “relax” (Conrad and Roth, 2007). In contrast, postural advice is commonly volitional, effortful, and positional (e.g., “stand up straight”; Harvard Medical School, 2017). However, volitional control (Teng et al., 2017; Russell et al., 2022) and even postural biofeedback (Baer et al., 2022) involve different brain circuitry from the brainstem structures that are engaged during postural support. Moreover, addressing posture positionally does not take into account the dynamic nature of tone. In general, current mainstream approaches to muscle tone are unlikely to fundamentally and optimally address its regulation.

### 7.2 Optimal tone regulation

Another obstacle to improving muscle tone concerns what constitutes “ideal” tone regulation. Healthy adults exhibit a range of tone properties, and it is unclear how muscle tone is optimally distributed, modulated and interconnected across the body. However, features can be identified which correlate with performance and pain. For instance, research showing that both hyper-and hypotonia lead to pain and motor impairment supports the idea that there is an ideal range for the level of tone. Axial tone also appears to be especially important for both performance and pain (Amiel-Tison and Grenier, 1983; Amiel-Tison, 2002), in particular through the engagement of deep muscles (Falla et al., 2004; Hodges and Danneels, 2019). Both the reduced modulation of tonic activity with pain (Claus et al., 2018; Gouteron et al., 2022) and the correlation of “suppleness” with recovery (Wernli et al., 2022) suggest that adaptability of muscle tone is beneficial. Theoretically, it might be important for tone to be both supportive and adaptable.

### 7.3 Insight from somatic practices

It may also be possible to gain insight into addressing muscle tone through disciplines that have empirically developed ways to influence it. In particular, many complementary and integrative somatic practices address muscle tension, and the implicit knowledge manifested in their ways of working might offer practical insight into tone regulation. Somatic practices aim to enhance performance and body–mind integration through awareness; they include the Alexander Technique, Feldenkrais Method, Tai Chi and Qigong. These disciplines also share a focus on tension and may reflect sophisticated ways of working with tone (Schmalzl et al., 2014). Of these, muscle tone has been studied most in the Alexander Technique, which has been found to increase adaptivity of axial tone (Cacciatore et al., 2011a; Cohen et al., 2015), but alterations in tone are also implicated in the Feldenkrais Method (Stephens et al., 2006). Changes in muscle tone may explain improvements in performance and pain resulting from these practices (Stephens et al., 2006; Little et al., 2008; MacPherson et al., 2013; Preece et al., 2016; Becker et al., 2021; Berland et al., 2022; Little et al., 2022; Hodges et al., 2024). While contextual factors such as expectations of improvement generally contribute to clinical benefit (Rossettini et al., 2018), pain reduction and alterations to tone and coordination from the Alexander Technique and Feldenkrais Method have exceeded that for control interventions (Little et al., 2008; Cacciatore et al., 2011a; MacPherson et al., 2013; Berland et al., 2022), suggesting a non-placebo mechanism. Many other complementary and integrative practices are consistent with addressing tension, but their effects on tone are not well studied, including Craniosacral Therapy, Hanna Somatics, Rolf Movement, Contact Improv, Total Motion Release for pediatrics (TMRTots), Somatic Experiencing and Jin Shin Jyusu. While somatic practices are distinct from one another and may alter muscle tone in different ways, similarities relevant to tone regulation include:

*Differentiation between subtle tensional states:* Distinctions are often made between states such as relaxed, released, engaged, tense, and responsive (Feldenkrais, 1964; de Alcantara, 1999; Cacciatore et al., 2005; Schmalzl et al., 2014; Osypiuk et al., 2018). It might be important to identify subtle aspects of tone in order to promote certain qualities. These different states may relate to the diversity of pathways that affect tone, and they may affect both excitability and tension.

*Mental attention to the body and space:* Somatic practices include attention and awareness on the body and space when addressing tension (Jones, 1976; Cacciatore et al., 2005; Schmalzl et al., 2014). These practices can also include subtle intentions regarding desired relations between body parts (de Alcantara, 1999), imagery (Cohen et al., 2015), as well as awareness of internal sensation (Mehling et al., 2011; Schmalzl et al., 2014). Verbal instructions based on the Alexander Technique influence axial tone and adaptability (Cohen et al., 2015), supporting the importance of high-level, attentional influences on muscle tone. Bodily and spatial attention in itself is salient for pain (Moseley et al., 2009; Torta et al., 2013; Lotze and Moseley, 2022) and may overlap with circuitry that influences tone (Gurfinkel and Levick, 1991; Gurfinkel et al., 1995a,b).

*Haptic touch:* Some somatic practices such as Feldenkrais, Craniosacral Therapy, TMRTots and the Alexander Technique incorporate light touch that aims to alter tone (Feldenkrais, 1964; Cacciatore et al., 2005; Brough et al., 2015). This may act by drawing attention to a body region or tensional relationship or might directly interact with the tone regulating circuitry at a lower level of the nervous system.

*Emphasis on involuntary regulation:* Many disciplines aim to prevent volitionally micromanaging and anticipating motor behavior (Bigé and Godard, 2019; Woods et al., 2020). This concept is described as “wu wei” in Tai Chi and “non-dong” in the Alexander Technique (de Alcantara, 1999). Thus, change originates from non-judgmental attention, awareness, intention and imagery, rather than from voluntary effort (Tang et al., 2022). For example, postural instructions based on the Alexander Technique are perceived as effortless in comparison with typical effortful modification of posture (Cohen et al., 2015). As tone pathways are largely distinct from those controlling voluntary action, such non-doing principles may be a way of targeting the relevant pathways to address the involuntary regulation of muscle tone.

*Whole body interactions:* The influence from one body region to another is widely acknowledged among complementary and integrative practices, and tension is often addressed remotely (Feldenkrais, 1964; de Alcantara, 1999; Schmalzl et al., 2014). This is consistent with tonic cross body interactions and may reflect overall organizing principles. Many somatic practices focus on axial tone including Qigong, Craniosacral Therapy, Feldenkrais and the Alexander Technique (Feldenkrais, 1964; de Alcantara, 1999; Brough et al., 2015).

*Resistance and compliance activities:* Activities involving resistance and compliance are ubiquitous in somatic practices. Examples include push hands and immovable arm in Tai Chi and Qigong (Chen et al., 2010), as well as many activities in Feldenkrais and the Alexander Technique. The desired resistance and compliance is described as “latent, automatic resistance and mobility” (de Alcantara, 1999), and is supported by measurements of increased compliance and resistance (Cacciatore et al., 2011a; Cacciatore et al., 2020). The involuntary nature of this resistance and compliance is consistent with an underlying state of “readiness” that facilitates adaptable tone, and may be closely related to excitability (Gurfinkel et al., 2006; Cacciatore et al., 2011a).

*Smooth, quasistatic movement*: Somatic practices often involve movement. However, the aim is not to perfect a particular movement trajectory but pertains to movement quality. Tai Chi, Qigong, Feldenkrais and the Alexander Technique all emphasize slow, smooth and reversible movement (de Alcantara, 1999; Liu and Frank, 2010; Russell, 2020). Slow movement decreases momentum and therefore may constrain postural support to be adaptive. For example, Alexander Technique teachers exhibit a prolonged and extremely smooth weight shift in sit-to-stand that is difficult to mimic (Cacciatore et al., 2011b; Cacciatore et al., 2014). The continuous shifting of weight to the feet may arise from increased adaptability that sensitively adjusts extensor muscle activity throughout the action (Cacciatore et al., 2020). Thus, the quasistatic nature of movement performed in somatic practices may act to increase the adaptability of muscle tone.

## 8 Discussion

Muscle tone reflects a foundational motor system that has the potential to be of widespread clinical importance. Research into muscle tone has been hampered by difficulty measuring and characterizing this multifaceted phenomenon, as well as by simplistic, reflex-focused models that neglected central regulation. The wide variation across various dimensions of this conservative, distributed, and adaptive system may underlie individual differences in performance and the predisposition for developing chronic pain. While the impact of muscle tone on pain is poorly understood, long-term pain-related alterations in tone are likely maladaptive. Thus, even when altered tone is not the primary cause of pain, addressing it may be clinically beneficial.

While its neurophysiology is still not well-understood, muscle tone results from a complex interconnected system that includes low-level feedback circuits regulated by descending tonic drive. This tonic drive arises from parallel, heterogeneous brainstem pathways and likely underlies the ability to centrally influence tone. While resting muscle tone is often passive, the active component may share circuitry with postural tone. Both phenomena likely reflect tonic drive and spinal excitability. However, postural tone is associated with increased reticulospinal drive (Mori et al., 1987; Zhang et al., 2024) and presumably has a greater dependence on feedback pathways that respond to load (Zelenin et al., 2013).

Muscle tone can affect musculoskeletal pain and motor performance, such as when tone is severely disrupted through neurological pathology, which represents an extreme case. Some evidence suggests that tone may also affect pain and motor performance more generally. This can occur mechanically, through the stiffness and compliance of the body, or by biasing neural processing through the excitability of low-level circuits. It is possible that the clinical importance of tone may stem from offering a window into this low-level excitably, rather than from tension *per se*.

Despite its complexity, mainstream practices often address muscle tone superficially, without accounting for the involuntary, interconnected and dynamic nature of the system. However, somatic practices appear to address tone in a way that is more consistent with the properties of the underlying system, which may explain the reductions in pain and improvements in performance that result from their use. These practices highlight the importance of bodily attention and awareness, avoiding volitional control, addressing tone non-locally, and differentiating between subtle tensional states.

While peripheral approaches, for example massage, vibration, and stretching, may be valuable for addressing muscle tone, it may also be important to address the central regulation of tone. This may be possible by influencing mental states, through awareness, attention or intention (Cohen et al., 2015).

An important difference between tone regulation and voluntary control may be the differential engagement of tonic and phasic neural pathways (Gurfinkel et al., 1999). While there are contrasting views on what constitutes voluntary control (Prochazka et al., 2000), researchers agree that it is deeply related to a sense of effort (Khachouf et al., 2017; Touroutoglou et al., 2020). Notably, effortlessness is emphasized in somatic practices (Feldenkrais, 1964; Tang et al., 2022) and postural alterations based on attention are associated with a lack of effort (Cohen et al., 2015). Attention is a strong modulator of motor behavior (Bogadhi et al., 2018) and attention to the body increases corticospinal excitability (Conte et al., 2007). Moreover, skilled bodily attention can increase activity within the spinal cord in a targeted way (Nejad et al., 2014); this may be an important mechanism of tone regulation, distinct from voluntary control.

Movement may also be an important tool for addressing muscle tone. For example, movement affects tone through neural integrators and serotoninergic descending drive, which is generally related to motor activity. In addition, constraining movement to be slow and smooth is commonplace in somatic practices and may promote the adaptability of tone (Cacciatore et al., 2014). Adaptability might also be enhanced through resistance and compliance activities, such as push hands in Tai Chi. Additionally, incorporating an effortless, “non-doing” approach through attention and awareness may help ensure that tone, rather than voluntary control, is being addressed.

While aspects of muscle tone remain poorly understood, sufficient data exist to form a conceptual model of tone regulation that is clinically beneficial. Future research should be directed toward better assessing the properties of tone and their neurological origin in human subjects, including developing integrated ways to characterize an individual’s distribution, adaptivity and cross-body interactions of tone. It will also be important to identify features of tone regulation that are salient for pain and performance. Another important research direction is to study the influence of somatic practices on tone, which may provide insight into healthy tone regulation and how tone can be changed. Questions include how somatic practices affect attention, awareness, interoception, and body representation, and how these processes affect tone and its associated neural circuitry.

## Data Availability

The original contributions presented in the study are included in the article/supplementary material, further inquiries can be directed to the corresponding author.
